# Quality and sources of food and water consumed by people with chronic kidney disease of unknown etiology in Sri Lanka: a systematic review

**DOI:** 10.1007/s40620-024-02174-5

**Published:** 2025-02-26

**Authors:** Nivedha Uthayarajan, K. L. T. D. Jayawardene, Ishanka Weerasekara

**Affiliations:** 1 Department of Medical Laboratory Science, Faculty of Allied Health Sciences, University of Peradeniya, Augusta Hill, Sri Lanka; 2 NSBM Green University, Homagama, Sri Lanka; 3 Institute of Health and Wellbeing, Federation University Australia, Melbourne, Australia; 4 Department of Health and Functioning, Faculty of Health and Social Sciences, Western Norway University of Applied Sciences, Bergen, Norway; 5 School of Health Sciences, The University of Newcastle, Callaghan, Australia; 6 School of Allied Health Science and Practice, Faculty of Health and Medical Sciences, The University of Adelaide, North Terrace, Australia

**Keywords:** BMI, Chronic kidney disease of unknown etiology, Food contamination, South East Asia, Water contamination

## Abstract

**Background:**

Prevalence data indicates that chronic kidney disease (CKD) affects approximately 15% of people worldwide, and chronic kidney disease of unknown etiology (CKDu) is highly prevalent in Sri Lanka. Food and water contamination are factors that were suggested as associated with CKDu. This systematic review aimed to summarize evidence on the patterns in quality and sources of food and water consumed by people with CKDu in Sri Lanka.

**Methods:**

MEDLINE, EMBASE, PsycINFO, and SLJOL databases were searched from inception to August 2024 for studies investigating the quality and sources of food and water consumed by the people with CKDu in Sri Lanka. Studies assessing children below 18 years, pregnant women and dialysis patients were excluded. Studies not specifically investigating CKDu were likewise excluded from the review. Two independent reviewers completed the screening, and the conflicts were resolved by consensus. Extracted data were presented as a narrative summary.

**Results:**

Of 1067 studies, 57 were eligible for the final analysis. Commonly investigated food sources were contaminated with heavy metals, while water sources were contaminated with heavy metals, toxic anions and cations, agrochemicals, fertilizers, herbicides, glyphosate, and aminomethylphosphonic acid (AMPA).

**Conclusion:**

Nephrotoxic heavy metals and fluoride contamination alter the quality of food and water, and pose high risks with regard to the kidney function of the people in Sri Lanka. Appropriate strategies to reduce the contamination of heavy metals, agrochemicals, and major ions that afftect the quality of water and food, should be implemented to lower the burden of CKDu in Sri Lanka.

**Graphical abstract:**

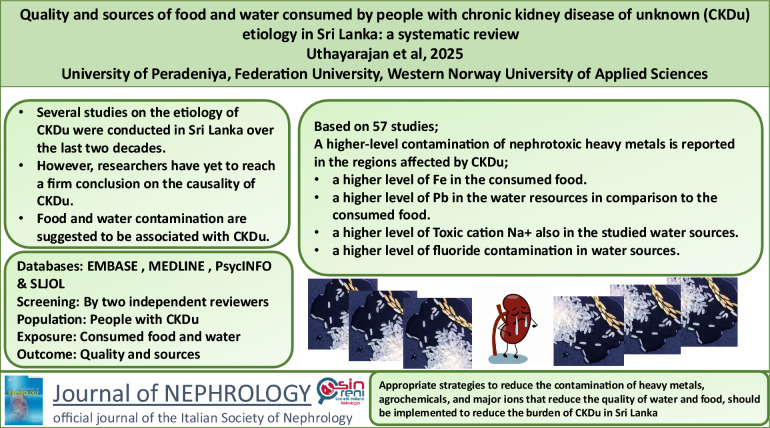

**Supplementary Information:**

The online version contains supplementary material available at 10.1007/s40620-024-02174-5.

## Introduction

Chronic Kidney Disease (CKD) is a major public issue with an estimated global prevalence of 15% [1]. It is defined as renal damage or impaired kidney function (Glomerular Filtration Rate (GFR) < 60 ml/min/1.73 m^2^ or Albuminuria ≥ 30 mg/24 h) for a period of three months or longer [2, 3]. Albuminuria, changes in renal imaging, hematuria/leukocyturia, persistent hydroelectrolytic disorders, histological changes in kidney biopsy, and previous kidney transplantation are reported as indicators of renal injury [4]. Diabetes, hypertension and glomerulonephritis are well-known causes of CKD [5–8]. CKD that is not linked to these etiologies is known as chronic kidney disease of unknown etiology (CKDu) [5, 8].

Since the 1990s, a novel type of CKD with no identifiable cause, i.e., CKDu, has been reported, thus increasing the number of nephrologic patients in rural areas of Sri Lanka [6]. Around 150,000 people are reported to have been affected by CKDu in Sri Lanka, with cases confirmed in North, North central, Eastern, and Uva provinces [9]. The prevalence of CKDu is high in the North central region of Sri Lanka, particularly in Medawachchiya, Girandurukotte, Kabithigollawa, Padaviya, Medirigiriya, Dehiattakandiya and Nikawewa regions [10].

CKDu has a direct impact on the lives of patients. As the disease progresses, the patients’ condition can adversely affect the family’s financial situation and overall well-being [11]. CKDu has no clear cause but is primarily seen in rural areas where ground water has generally been the primary source of drinking water [12]. The synergistic effects of fluoride, hardness, and heavy metals such as aluminum and cadmium also pose a health risk to humans [13]. Heavy metal intake via food and water has also been reported as a possible cause for CKDu [1, 14–16]. Therefore, it is important to assess the food and water quality in the affected CKDu endemic areas in order to reduce the burden of CKDu.

Several studies on the etiology of CKDu were conducted in Sri Lanka over the last two decades [17]. However, researchers have yet to reach a firm conclusion on a specific cause or causes of CKDu [17]. Food and water contamination are suggestive of factors associated with CKDu [18–21]. Hence, there is a crucial need for a systematic review to compile and provide a comprehensive summary of the findings on sources and quality of food and water among people with CKDu in Sri Lanka.

This systematic review aims to summarize the evidence on the patterns in quality and sources of food and water consumed by people with CKDu in Sri Lanka. The specific objectives are to identify patterns in quality and sources of food among people with CKDu in Sri Lanka and to identify the areas of inconsistency and gaps in the evidence on the quality and sources of food and water among people with CKDu in Sri Lanka.

## Materials and methods

The Preferred Reporting Items for Systematic Reviews and Meta Analyses (PRISMA) 2020 guideline for reporting systematic reviews was followed during this systematic review [22]. The protocol was published in the International Prospective Register of Systematic Reviews (PROSPERO) on 10 May, 2022 (CRD42022323219). This study collates all the available evidence from inception to the date of search, on the research outcomes of interest of people with CKDu in Sri Lanka. CKD that has no clear cause, referred to as CKDu, was investigated through this systematic review. Studies that assessed sources and quality of consumed food and water among adults (18 years or over) with CKDu in Sri Lanka were included in this systematic review.

The following databases were searched from inception to August 2024; MEDLINE, EMBASE, PsycINFO, and Sri Lankan Journals Online Database (SLJOL). The last search date was 21August, 2024. A gray literature search was conducted in ResearchGate, Google Scholar, and also by citation tracking of the relevant papers. Key words related to ‘chronic kidney disease’, ‘food and diet’, ‘water’, and ‘Sri Lanka’ were used. The language was restricted to English. The MEDLINE search strategy is in Appendix 1.

The yielded search was screened independently by two researchers at the title and abstract stage, followed by full text screening. Pre-defined inclusion and exclusion criteria were formed based on the Participant-Intervention-Comparison and Outcome (PICO) criteria. Cross-sectional studies investigating Sri Lankan adults (18 years or over) with CKDu for their food intake and diet, source and water quality and nutritional status were included. Studies assessing children, pregnant women, dialysis patients, surgical patients, trauma patients, and patients with other kidney diseases, and studies on animals were excluded. Studies exploring CKD but not specifically investigating CKDu were excluded from the review. Studies assessing morphological and clinical characteristics of CKDu, and study designs such as case-studies, case series, conference articles, commentaries, research notes, thesis papers, systematic reviews, scoping reviews, literature reviews, editorials, and studies that were not peer-reviewed were also excluded. Data from each included publication, including the type of study, study population (location, age, gender), sample size, food/water source, trace element levels, major cation/anion levels, agrochemical/fertilizer levels in water/food, physicochemical characteristics of water, and outcomes related to the study objectives of the study were extracted by a single researcher.

Methodological quality was assessed by one reviewer using the quality assessment tool for observational cohort and cross-sectional studies. This tool assesses the methodological quality of included studies in the following domains: research question or objective clearly stated, study population clearly defined and specified, all the subjects/samples selected from the same or similar population, pre-specified inclusion and exclusion criteria, sample size justification, exposure(s) of interest measured prior to the outcome(s), sufficient time frame, different level of exposures as related to the outcomes are examined, exposure measures clearly defined, valid, and reliable, exposure(s) measured more than once over time, outcome measures clearly defined, valid, and reliable, outcome assessors blinded to the exposure status, Follow-up after baseline ≤ 20%, and adjusted for potential confounding variables. The reviewers assigned a score of 2 (cannot determine/not applicable/not reported) or 1 (yes) or 0 (no), for each item.

A narrative synthesis of the literature was carried out by presenting summary tables and graphs to address the objectives. There were no outcomes found to be eligible for a meta-analysis due to higher heterogeneity of the study samples i.e. collected food samples ranged from rice, legumes, bread, animal sources food, eggs, freshwater fish, fruit, and vegetables, to black tea samples.

## Results

**Fig. 1:**
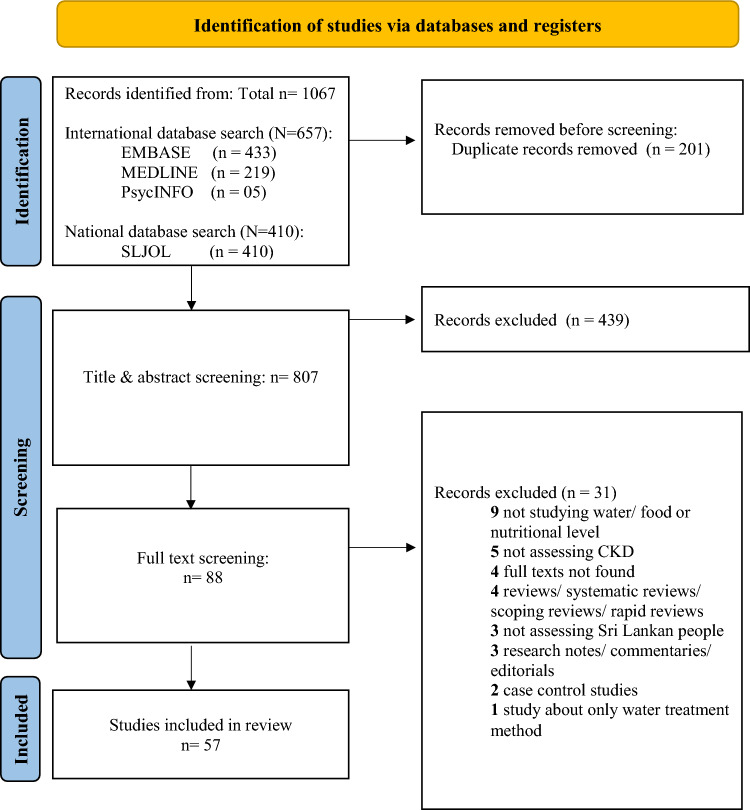
PRISMA flowchart (n = Number of studies)

The initial search yielded a total of 1067 studies (EMBASE, 433; MEDLINE, 219; PsycINFO, 5; SLJOL; 410). After removing duplicates, 807 titles and abstracts were screened, after which 88 full texts were obtained and further analyzed. Data extraction of the final, eligible 57 studies was undertaken by one researcher (Fig. 1).

A summary of the findings of this systematic review are presented as follows; (1) sources and quality of the food consumed by the people with CKDu in Sri Lanka (Table 1), (2) sources and quality of the water consumed by the people with CKDu in Sri Lanka (Table 2), (3) Body mass index (BMI) of the people with CKDu in Sri Lanka (Appendix 2), and (4) research gaps, weakness, inconsistencies, and future research suggestions (Table 3). These findings represent Padaviya, Rajanganaya, Medawachchiya, Nikawewa, Huruluwewa, Ullukkulama, Kumbichchankulama, Karapikkada, Alankulama, Thuruwila wewa, Thirappane, Maradankadawala, Rambewa, Nuwaragampalatha Central, and Galnewa in Anuradhapura district, Medirigiriya in Polonnaruwa district, Sewanagala and Wellawaya in Monaragala district, Girandurukotte, Mahiyanganaya in Badulla district, Dambulla and Wilgamuwa in Matale district, Hambantota district, Dehiaththakandiya in Ampara district, Kandy district, Vavuniya districts, Thunukkai in Mullaitivu district, Matara and Galle districts in Sri Lanka.

**Table 1:** Sources and quality of the consumed food by the people with CKDu in Sri Lanka

First author & year Geographical location Study design	Food sources		Toxic elements in food	Major anions in food	Other compounds in food	Findings
Wanigasuriya et al. (2008) [23] Medawachchiya, Padaviya, Rajanganaya in NCP Survey	Maize (*Zea mays*), raw and parboiled rice (*Oryza sativa*), and five pulses and legumes [mung, cowpea, kurukkan, soya bean and undu]		NR	NR	OA	Regular consumption of OA at levels present in the food items tested was unlikely to be a direct cause of CKDu in the NCP
Chandrajith et al. (2010) [37] E: Girandurukotte, Nikawewa, Medawachchiya NE: Huruluwewa Cross- sectional study	Rice samples		As, Cd, Pb, Al, Cu, Zn, Mn, Se	NR	NR	Cd was not associated with CKDu occurrence
Jayatilake et al. (2013) [14] E: Anuradhapura, Polonnaruwa and Badulla NE: Hambantota Cross-sectional study	Rice, pulses, vegetables; leafy vegetables, coconut, yams and roots (such as kohila, lotus), and freshwater fish		As, Cd, Pb	NR	NR	Cd exposure was a risk factor for CKDu
Jayalal et al. (2015) [15] CKD endemic areas Survey	Rice samples, and vegetables		Cd	NR	NR	Excess Cd exposure via food in the study areas
Dhanapala et al. (2015) [24] Padaviya in Anuradhapura district Survey	Rice samples		Fe, Cu	NR	NR	Relative risk of dietary contaminants was greater than 1
Levine et al. (2016) [25] Medawachchiya, Medirigiriya in NCP Survey	Rice sample, and freshwater fish sample		As, Cd, Cr, Cu, Fe, Hg, K, Mg, Mn, Na, Ni, Pb, Se, Sn, Zn, Ca	NR	NR	Rice and freshwater fish were unlikely to cause CKDu
Herath et al. (2018) [26] All districts Cross-sectional, survey	Rice; polished and unpolished rice samples		As, Cd, Pb, Cr	NR	NR	As, Cd, Pb, and Cr in rice were not associated with the occurrence of CKDu
Jayalal et al. (2019) [27] Medawachchiya, Padaviya in Anuradhapura district Survey	Rice, cereal (non- rice), inland fish, egg, fats and oil, vegetables; fruit/flower vegetables, leafy vegetables, legume vegetables, root and tuber vegetable and fruits		As, Cd, Pb	NR	NR	Chronic exposure of Pb and Cd was of public health concern, while As exposure was within the safe limit
Nanayakkara et al. (2019) [28] NCP Survey	Rice samples		As, Cd, Pb	NR	NR	Trace metals; Cd and As toxicity was not an etiological factor of CKDu
Jayalal et al. (2020) [30] Padaviya in Anuradhapura district Cross-sectional study	Rice samples		Cd, Pb, Hg	NR	NR	Cd and Pb levels might be associated with adverse health impacts
Fernando et al. (2020) [29] Medawachchiya area; Mahadivulwewa, Puhudivula, Kirigollewa, Unagaswewa, Karanbankulama**,** Ampara Cross-sectional	Rice samples; raw rice (RC), and cooked rice (CR)		Cd, Pb, Cr	NR	Total Phenolic Total flavonoid Total proanthocynadine Free amino acid	Rice flavonoid content might be an important variation factor of CKDu prevalence Proanthocynadine and free amino acid were not significant Significant negative correlation between flavonoid content and Cd and Cr contents in rice grains Chronic intake of Cd and Cr may result in kidney failure
Weerasekara et al. (2022) [31] Padaviya in Anuradhapura district Survey	Fish samples; Nile tilapia and butter catfish		As, Cd	NR	NR	Cd and As contents of these fishes were highly unlikely to pose non-carcinogenic human health risks to moderate level consumers
Chandrajith et al. (2021) [32] Girandurukotte in Badulla district Cross- sectional	Black tea samples; loose tea, branded/ packed tea, tea from TRI sites, MMU black tea, black tea and oven-dried tea, and tea infusion		NR	F^–^〹	NR	Excessive tea consumption was discovered to be an additional risk factor for excessive F^–^ intake
Kulathunga et al. (2021) [33] Dambulla Survey	Vegetables; root vegetables, stem vegetables, leafy vegetables, fruits vegetables, and legume vegetables		As, Cd, Pb, Cu, Zn, Mn,	NR	NR	Significant variation of Mn, Co, Cu, Zn, As, Cd and Pb concentrations among the vegetables No/ minimal adverse health risks caused by Mn, Co, Cu, Zn, As, Cd and Pb levels present in the vegetables
Kulathunga et al. (2021) [33] Medirigiriya in Polonnaruwa district Cross- sectional, survey	Rice, vegetables, animal source food, and other food items (bread, pulses)		As, Cd, Pb, Co, Mn, Cu, Zn, Se, V	NR	NR	Pb can inflict adverse health effects, whereas the potentially dangerous effects caused by Cd and As are minimal
Edussuriya et al. (2022) [35] Dehiattakandiya, Girandurukotte and Sewanagala Cross-sectional	Black tea samples	Na^+^,Mg^2+^, K^+^, Ca^2+^		F^–^Cl^–^SO_4_^2–^PO_4_^3–^	NR	Tea infusions made with groundwater from CKDu endemic locations had higher levels of fluoride and major cations
Wanigasuriya et al. (2011) [75] Dehiattakandiya, Girandurukotte and Sewanagala Cross-sectional	Rice samples	Na^+^,Mg^2+^, K^+^, Ca^2+^		F^–^, Cl^–^SO_4_^2–^, PO_4_^3–^	NR	F^−^, Cl^−^, and Na^+^ content in raw rice samples collected from CKDu endemic areas were significantly higher whereas SO_4_^2−^ and PO_4_^3−^ were lower when compared to the raw rice samples collected from CKDu non-endemic areas
Lockwood et al. (2023) [36] Medawachchiya in Anuradhapura district of NCP Cross-sectional	Rice samples (Suwadel, Kalu Heenati, Rathal, Madathwalu, Siyapathal (Pachchaperumal), Swanjatha, Ran Kahawanu)		Cr, Co, Ni, As, Cd, Pb, Mn, Fe, Cu, Zn, Se, Mg, K, Ca, Al	NR	NR	The concentration of Cd was above Australian guidelines in one sample; no other nephrotoxic metals were detected in unsafe concentrations

*Al* Aluminium, *As* Arsenic, *Ca* Calcium, *Ca*^*2+*^ Calcium ion, *Cd* Cadmium, *CKDu* Chronic kidney disease of uncertain etiology, *Cl−* Chloride, *Co* Cobalt, *Cr* Chromium, *CR* Cooked rice, *Cu* Copper, *E* Endemic area, *F−* Fluoride, *Fe* Iron, *Hg* Mercury, *K* Potassium, *K*^*+*^ Potassium ion, *Mg* Magnesium, *Mg*^*2+*^ Magnesium ion, *MMU black tea* Miniature manufactured ungraded black tea, *Mn* Manganese, *Na* Sodium, *Na*^*+*^ Sodium ion, *NCP* North Central province, *NE* Non-endemic area, *Ni* Nickle, *NR* Not reported, *OA* Ochratoxin A, *Pb* Lead, *PO*_*4*_^*3–*^ Phosphate, *RC* Raw rice, *Se* Selenium, *SL* Sri Lanka, *Sn* Tin, *SO*_*4*_^*2–*^ Sulphate, *TRI* Tea Research Institute, *Zn* Zinc

**Table 2:** Sources and quality of the consumed water by the people with CKDu in Sri Lanka

First author & year Geographical location Study design	Water source	Toxic elements/ cations in water	Major anions/ compounds in water	Agro- chemicals / fertilizer in water	Physio chemical characteristics	Findings
Wanigasuriya et al. (2007) [23] Anuradhapura district Survey	Home wells, Pipeborne, and field wells	NR	NR	NR	NR	CKDu is an environmentally-induced disease
Chandrajith et al. (2010) [37] E: Giradurukotte, Nikawewa, Medawachchiya, Padaviya NE: Huruluwewa, Wellawaya Cross- sectional	Wells	Cd, U, Al, Mn^2+^, Na^+^, K^+^, Ca^2+^, Mg^2+^, Fe^3+^	NO3^–^ -N, F^–^, Cl^−^, NO_3_^−^, SO_4_^2−^, PO_4_^3−^	NR	pH EC Alkalinity Hardness	Fluoride alone is not a causative agent of CKDu Fluoride toxicity depends strongly on Na^+^ and Ca^2+^ activities
Chandrajith et al. (2010) [37] E: Girandurukotte, Nikawewa,, Medawachchiya NE: Huruluwewa Cross-sectional survey	Dug wells, and deep wells	As, Cd, Pb, Al, Cu, Zn, U, Ni, Li, B, Mn, Co, Se, Rb, Sr, Mo	F^–^	NR	NR	Cd was not associated with CKDu No single geochemical or biogeochemical parameter was associated with CKDu
Jayasekara et al. (2012) [5] Padaviya, Nikawewa NR	Shallow wells, tube wells and water reservoirs	NR	NR	NR	NR	Possible environmental factor for CKDu occurrence is water and the aetiological agent is water soluble
Jayatilake et al. (2013) [14]] E: Anuradhapura, Polonnaruwa, Badulla NE: Hambantota Cross-sectional	Ground wells, tube wells, natural springs, irrigation canals, and reservoirs	As, Cd, Pb	NR	NR	NR	Multiple agents associated with CKDu pathogenesis
Nanayakkara et al. (2013) [38] Medawachchiya Girandurukotte Survey	Dug wells, tube wells, surface water sources and treated water	As, Cd, Pb	NR	NR	NR	Absence of nephrotoxic heavy metal contamination in drinking water
Jayasekara et al. (2014) [43] Mahiyanganaya, Girandurukotte, and Nikawewa Cross- sectional	Natural springs and shallow wells	NR	NR	NR	NR	High possibility of CKDu occurrence due to drinking water sources
Jayalal et al. (2015) [15] CKD endemic areas Survey	NR	Cd	NR	NR	NR	SL people in endemic areas exposed to excess levels of Cd from water
Dhanapala et al. (2015) [24] Padaviya in Anuradhapura Survey	Dug wells and tube wells	Fe, Cu	NO_3_^—^N, F^−^	Organic phosphorous	pH EC TDS Temperature Total Hardness	pH, EC and TDS in well water were below the SLPWL, while NO_3_^−^ -N, hardness and F^−^ values exceeded the SLPWL in some wells Both Fe and Cu concentrations in well water were lower than the PMTDI of WHO
Diyabalanage et al. (2015) [42] NCP Cross-sectional	Rivers, and reservoirs	As, Co, Cu, Fe, Mn, Ni,Cr, Se, Zn, Mo	NR	Agrochemicals and fertilizers	NR	Trace metals impact the water quality of the upper Mahaweli catchment. However, it is still within the recommended levels
Rango et al. (2015) [18] Anuradhapura, Polonnaruwa, Kandy, Badulla, Ampara, Vavuniya Survey	Dug wells, tube wells, springs, and pipe water	As, Cd, Pb, U	F^−^	NR	pH Temperature EC Eh values	Alcohol and cigarette consumption were more highly correlated with CKDu than the nephrotoxic elements such as As, Cd, U, and Pb
Siriwardhana et al. (2015) [39] Medawachchiya in NCP Survey	Wells, springs, and tubewells	NR	NR	NR	NR	CKDu is likely to be associated with consumption of untreated well water
Wasana et al. (2015) [13] NCP Survey	Dug wells, tube wells, and spring	As, Cd, Ca, Mg, Al	F^−^	NR	Hardness	Synergistic effect of hardness, F, Al and Cd with the incidence of CKDu
Levine et al. (2016) [25] Medawachchiya, Medirigiriya in NCP Survey	Sediment-free drinking water	As, Cd, Cu, Fe, Mn, Pb, Ni, Cr, Zn, Se, Mg, Ca, Hg, Sn, Na	F^−^	NR	Hardness	No single hydro-geochemical parameter directly related to CKDu
De Silva et al. (2017) [44] Medawachchiya in Anuradhapura District NR	Dug wells	NR	NR	NR	NR	Direct correlation between the use of unprotected private dug wells and CKDu occurrence can be seen
Edirisinghe et al. (2017) [47] Dehiaththakandiya, Medirigiriya, Padaviya, Nikawewa in NCP Cross-sectional	Shallow GW (dug wells), deep GW (tube wells), surface water (rivers, tanks, canals and streams), and rain water	As, Cd, Pb	NR	NR	δ^18^O and δ^2^H	The origin, recharge mechanism and flow pattern of GW, and geological conditions were associated with CKDu in the dry zone of SL Anthropogenic inputs on water chemistry had no significant effect for the CKDu
Herath et al. (2017) [45] All districts Survey	Wells	As, Mn, Al	F^−^, NO_3_^−^	NR	pH Hardness (CaCO_3_)	Well water contaminated with high concentrations of F^−^, NO_3_^−^, Al, As, and Mn, in addition to high hardness, causing potential health risk for the people
Wickramarathna et al. (2017) [46] Girandurukotte, Wilgamuwa and Nikawewa Survey	Drinking water wells, irrigation reservoirs (tanks), and surface drains	As, Cd, Cu, Fe, Al, Mn, Pb, Co, Ni, Cr,Zn, Se, Mo, Sr, Ba, Li, B, Na^+^,K^+^,Ca^2+^, Mg^2+^	HCO_3_^−^ Cl^−^, F^−^, NO_3_^−^, PO_4_^3−^, SO_4_^2−^	Hardly use fertilizers during monsoon	pH EC Alkalinity Hardness	No association of studied trace elements with CKDu Fluoride and hardness were associated with CKDu
Gunarathna et al. (2018) [49] Medawachchiya in Anuradhapura Cross-sectional	Shallow wells, and lakes	Ca, Mg, Al	NR	Glyphosate AMPA	pH Hardness Temperature	Marginal impact of glyphosate and AMPA on CKDu
Herath et al. (2018) [26] All districts Cross-sectional survey	Dug wells, and tube wells	As, Cd, Pb, Cr	NR	NR	NR	Cd, As, Pb and Cr in well water are not possible causes of CKDu
Makehelwala et al. (2018) [53] NCP Survey	Shallow ground water	NR	NR	2,4-D PCP PRP 3,4-DCA	DOC COD_Mn_ COD_Mn_/DOC ratio	NR
Siriwardhana et al. (2018) [39] Medawachchiya in Anuradhapura Interventional study	Bottled water and habitual drinking water	NR	NR	NR	NR	Habitual drinking water is positively associated with CKDu progression
Wanasinghe et al. (2018) [50] Maradankadawala Thirappane areas in Anuradhapura Survey	Wells and tanks	As^3+^, Pb^2+^,Cd^2+^, Na^+^,Ca^2+^, Mg^2+^	SO_4_^2−^, PO_4_^3−^, Cl^−^, NH_4_^+^-N NO_3_^−^-N	NR	pH EC Alkalinity TDS Turbidity	NR
Kafle et al. (2019) [52] Mullaitivu, Vavuniya, Anuradhapura, Trincomalee, Polonnaruwa, Kurunegala, Matale, Badulla, Ampara, Monaragala Survey	Household wells, agro-wells or springs	NR	NR	NR	NR	Historical groundwater reliance was consistently higher in affected households than in non-affected households, across districts
Makehelwala et al. (2019) [53] Padaviya NR	Groundwater and reservoir	NR	NR	NR	Higher surface C composition and lower O for HDOC than for SHA	NR
Nanayakkara et al. (2019) [28] NCP Survey	Dug wells, tube wells, surface water sources and treated water	As, Cd, Cu, Mn, Pb, Ni, Co, Cr, Zn, Se, Sr, Ba, Al, U, Rb, K, Mg, Ca	NR	NR	NR	As, Cd and Al were not associated with CKDu pathogenesis
Babich et al. (2020) [1] E: Medirigiriya in Polonnaruwa, and Padaviya in Anuradhapura NE: Matara and Galle Cross-sectional	Reservoirs, drinking well, and rice field water	As, Cd, Co, Cu, Fe, Mn, Pb, Ni, Cr, Sb, Se, V	NR	Glyposhate	NR	Association of heavy metals in drinking water, with impaired kidney development
De Silva et al. (2020) [19] Medawachchiya, Kebithigollewa in Anuradhapura, Medirigiriya in Polonnaruwa NR	Dug wells and tube wells	NR	NR	NR	NR	Changing over to RO water has had positive health outcomes and has reduced the progression of CKDu
Dissanayake et al. (2020) [54] Moneragala district Cross-sectional	Dug wells, tube wells, and surface water from reservoirs and rivers	Na^+^, Mg^2+^,K^+^, Ca^2+^	F^−^, Cl^−^ NO_3_^−^,PO_4_^3−^, SO_4_^2−^, HCO_3_^−^	NR	pH DO TDS EC Hardness	Due to high fluoride content in CKDu patients’ wells in CKD/CKDu endemic areas, the quality of water is poor
Fernando et al. (2020) [29] Medawachchiya in Anuradhapura Cross-sectional	Well water (Cooking water)	Cd, Pb, Cr	NR	NR	NR	Presence of Cd, Pb, and Cr in cooking water is not sufficient to increase heavy metal content in cooked rice
Gobalarajah et al. (2020) [11] Thunukkai division in Mullaitivu Survey	Dug wells and tube wells	As, Cd, Mg^2+^, Ca^2+^,Na^+^, K^+^	NO_3_^−^, F^−^, Cl^−^, PO_4_^3−^, SO_4_^2−^	NR	pH EC Salinity TDS Turbidity Total hardness Alkalinity	High ionic content leading to higher EC, salinity, total dissolved solids and total hardness levels compared to SL standards could be seen Significant correlation of phosphate, TDS and As content with the CKDu in the study area
Imbulana et al. (2020) [55] Anuradhapura District HR: Rambewa, Medawachchiya, Nuwaragampalatha Central LR: Galnewa and Thirappane Survey	Dug wells, and tube wells	Na^+^, K^+^,Ca^2+^, Mg^2+^,Total Fe, NH_4_^+^	F^−^, Cl^−^, SO_4_^2−^, NO_3_^−^	No contamination of agrochemicals	pH Total alkalinity EC TDS Hardness DOC level Total coliform	Association of higher concentrations of alkalinity, hardness, TDS, and major ions ( Ca^2+^, Mg^2+^, Na^+^, K^+^, Cl^−^ and SO_4_^2−^) with the occurrence of CKDu No association of CKDu with fluoride and DOC Reduction of the incidence of CKDu with RO-treated water could be seen
McDonough et al. (2020) [57] Medawachchiya in Anuradhapura district Pilot study	Wells and springs	As, Ba,Ca, Cl, Cr, Cu,K, Mg, Na, Ni,U, Zn	F, NO_3_^−^, P	NR	Total of 58 unique phyla categories DO ^13^C_DIC_ ^13^C_POC_	Presence of cyanotoxin-producing Microcystis in drinking water may be positively associated with CKDu
Nanayakkara et al. (2020) [58] Girandurukotte, Medawachchiya in NCP Survey	Dug wells, tube wells, surface water sources and treated water	NR	F^−^	NR	NR	Association of elevated fluoride concentrations with CKDu
Nikagolla et al. (2020) [59] Mahiyanganaya, Padaviya, Medawachchciya, Rambewa Survey	Shallow ground water wells, deep wells, and springs	As, Cu, Mn, Fe, Ni, Zn, Si, Rb, Sr,Cr, Se,U, Na^+^,K^+^,Ca^2+^, Mg^2+^	F^−^, Cl^−^, SO_4_^2−^, NO_2_^−^, NO_3_^−^, HCO_3_^−^, CO_3_^2−^	NR	DO pH EC δ^2^H δ^18^O δ^13^C_DIC_	No association of metals with CKDu High fluoride concentration was not the only factor associated with CKDu occurrence
Chandrajith et al., 2021 [32] Girandurukotte area Cross-sectional	Ground water samples	NR	F^–^	NR	Hardness	Teas brewed using water with elevated F^–^ contents produce tea infusions with elevated F^–^ levels
Indika et al. (2021) [60] Anuradhapura, Polonnaruwa Cross-sectional	Feed water; ground water, and RO treated water	As^3+^,Cd^2+^,Mn^2+^, Fe^2+^,Cu^2+^, Zn^2+^, Cr^3+^, Hg^2+^, Ca^2+^, Mg^2+^,Na^+^, K^+^, Li^+^, Sr^2+^,Ba^2+^	F^−^, Cl^−^ Br^−^,NO_3_^−^, PO_4_^3−^, SO_4_^2−^	NR	pH EC Hardness Alkalinity	Most RO systems produced drinking water with high quality
Liyanage et al. (2021) [21] Monaragala district Survey	Dug wells, and tube wells	As, Cd, Cu, Fe, Mn, Pb, Ni, Co, Cr, Zn, Se, Sr, Ba, Li, Al, V, Rb, Na, K,Mg, Ca	F^−^	NR	pH EC Hardness	Heavy metals such as As, Cd, Ni, Co, Zn, and Pb not associated with CKDu Association of increased ionicity with CKDu occurrence Excess fluoride and water hardness might be associated with CKDu
Chandrajith and Diyabalanage (2022) [61] Girandurukotte, Dehiattakandiya and Sewanagala Cross-sectional	Wells	Na, K,Ca, Mg, Li, Al, Cr, Mn, Fe,Co, Ni, Cu, Zn, As, Se, Rb, Sr, Cd, Ba, Pb	F^−^, Cl^−^, NO_3_^−^, PO_4_^3−^, SO_4_^2−^, NO_3_^−^	NR	pH EC TDS Alkalinity Hardness	Dry zone ground water often has higher levels of dissolved solids. Excess fluoride, hardness, and Arsenic were among the many concerns raised
Hu et al. (2022) [62] Anuradhapura and Polonnaruwa in NCP Cross-sectional	Dug wells, and tube wells	Na^+^, K^+^, Mg2^+^, Ca2^+^	F^−^,Cl^−^, Br^−^, NO_3_^−^ PO_4_^3−^, SO_4_^2−^	NR	pH EC TDS Alkalinity Hardness TOC DOM	The mean concentrations of hardness and fluoride for both dug and tube wells exceeded limits of the Sri Lankan Drinking Water Standard. The average concentration of TOC in both aquifers is higher than 5.0 mg/L
Hu et al. (2023) [64] 29 DS divisions in Anuradhapura and Polonnaruwa in NCP Cross-sectional	Dug wells, tube wells and springs	Mn, Zn, Al, Fe, Cu, Ni, As, Pb, Cr, Ti, Cd, Na^+^, K^+^, Mg2^+^, Ca2^+^	F^−^, Cl^−^ Br^−^, NO_3_^−^, SO_4_^2−^,HCO_3_^−^	NR	pH EC TDS Temperature	High hardness in ground water dominated by anion such as HCO_3_^−^, and cations such as Na^+^ and Ca^2+^ Water quality of Polonnaruwa is generally better than that of the Anuradhapura
Shi et al. (2023) [65] Girandurukotte, and Dehiattakandiya Cross-sectional	Ground water samples	Na, K, Ca, Mg, Si, Al, As,Fe, Mn, Br, Sr, Pb, Zn, Ba, Se, Cr, Cd, Li	F^−^, Cl^−^ NO_3_^−^, PO_4_^3−^, SO_4_^2^	Calcite Fluorite Quartz Silica Talc Sepiolite	pH EC Depth TDS ORP DO Total Hardness	Groundwater samples with CKDu showed significantly higher Si and F^−^ contents, perhaps contributing to the condition
Sandanayake et al. (2023) [63] Girandurukotte, Dehiattakandiya and Sewanagala Cross-sectional	Dug wells, tube wells, streams, and lakes	Na^+^, K^+^, Mg2^+^, Ca2^+^	F^−^, Cl^−^ Br^−^, NO_3_^−^, PO_4_^3−^, SO_4_^2^	NR	pH EC TDS Alkalinity Hardness	No significant difference was observed in fluoride levels between CKDu endemic and non-endemic areas

*Al* Aluminium, *AMPA* Aminomethylphosphonic acid, *As* Arsenic, *As*^*3+*^ Arsenic ion, *B* Boron, *Ba* Barium, *Ba*^*2+*^ Barium ion, *Br*^*−*^ Bromide, *Ca* Calcium, *Ca*^*2+*^ Calcium ion, *Cd* Cadmium, *CKDu/ CKDue* Chronic kidney disease of uncertain etiology, *Cl*^*−*^ Chloride, Co Cobalt, *CO*_*3*_^*2−*^ Carbonate, *COD* Chemical oxygen demand, *Cr* Chromium, *Cr*^*3+*^ Chromium ion, *Cu* Copper, *Cu*^*2+*^ Cupric ion, *2,4-D* 2,4-dichlorophenoxyacetic acid, *3,4-DCA* 3,4-dichloroaniline, *DO* Dissolved Oxygen concentration, *DOC* Dissolved organic carbon, *DOM* Dissolved Organic Matter, *E* Endemic area, *EC* Electrical conductivity, *Eh value* Redox potential value, *ERP *Eppawala rock phosphate, *F*^*−*^ Fluoride, *Fe* Iron, *Fe*^*3+*^ Ferric ion, *Fe*^*2+*^ Ferrous ion, *GW* Ground water, *HCO*_*3*_^*−*^ Bicarbonate, *Hg*^*2+*^ Mercuric ion, *HR* High risk area, *K* Potassium, *K*^*+*^ Potassium ion, *Li* Lithium, *Li*^*+*^ Lithium ion, *LOD* Lower detection limit, *LR* Low risk area, *Mg* Magnesium, *Mg*^*2+*^ Magnesium ion, *Mn* Manganese, *Mn*^*2+*^ Manganese ion, *Mo* Molybdenum, *mV* Millivolt, *Na* Sodium, *Na*^*+*^ Sodium ion, *NCP* North Central province, *NE* Non-endemic area, *NH*_*4*_^*+*^*-N* Ammonium nitrogen, *Ni* Nickle, *NO*_*3*_^*−*^ Nitrate, *NO*_*3*_^*−*^*-N* Nitrate-nitrogen, *NO*_*2*_^*−*^ Nitrite, *NR* Not reported, *Pb* Lead, *Pb*^*2+*^ Lead ion, *PCP* Pentachlorophenol/ pesticide, *PMTDI* Provisional Maximum Tolerable Daily Intake, *ppm* Parts per million, *PRP* Propanil, *Rb* Rubidium, *RO* Reverse osmosis, *Sb* Antimony, *Se* Selenium, *Si* Silicon, *SiO*_*2*_ Silica, *SL* Sri Lanka, *SLPWL* Sri Lankan Permissible Water Limit, *SO*_*4*_^*2−*^ Sulphate, *Sr* Strontium, *Sr*^*2+*^ Strontium ion, *SW* Surface water, *TDS* Total dissolved solids, *TOC* Total Organic Carbon, *U* Uranium, *V* Vanadium, *WHO* World Health Association, *Zn* Zinc, *Zn*^*2+*^ Zinc ion

### Sources and quality of food intake among people with CKDu in Sri Lanka

Eighteen studies explored the quality of food and diet of Sri Lankans with CKDu [10, 14, 15, 20, 23–31, 33–36]. Among these, 11 were conducted in the Anuradhapura district and four were carried out in the Polonnaruwa district in the North Central province, five studies in the Badulla district, three studies in the Ampara district, two studies in all 25 districts, and Monaragala district, one study each in Matale and Hambantota districts. Food samples such as rice [polished raw and unpolished raw, and parboiled rice (*Oryza sativa*)], maize (*Zea mays*), five pulses and legumes [mung, cowpea, kurukkan, soya bean and undu], bread, animal source foods, eggs, fresh water fish [Nile tilapia and butter catfish (*Oreochromis niloticus*)], cows’ milk, fats and oil, lotus rhizome (*Nelumbo nucifera*), fruit and vegetables [e.g. stem, leafy vegetables, coconut, yams and roots (e.g. kohila, lotus)], and black tea samples [loose tea, branded/packed tea, tea from tea research institute (TRI) sites, miniature manufactured ungraded (MMU) black tea, black tea, oven-dried tea, and tea infusions] were assessed in the included studies. The authors mainly looked for Arsenic (As), Cadmium (Cd), Lead (Pb), Aluminum (Al), Chromium (Cr), Cobalt (Co), Copper (Cu), Iron (Fe), Manganese (Mn), Zinc (Zn), Selenium (Se), Mercury (Hg), Tin (Sn), Potassium (K), Sodium (Na), Magnesium (Mg), Nickel (Ni), Calcium (Ca), and Vanadium (V) heavy metal concentrations in the diets. Cadmium was the most frequently reported heavy metal contaminant, as observed in 14 studies [10, 14, 15, 20, 24–31, 33, 36]. The second was Lead [10, 20, 25–30, 33, 36] and the third most common contaminant was Arsenic [10, 20, 25–28, 31, 33, 36]. In addition, Mn, Zn, Cu, Fe, Cr, Se, Co, Al, Hg, Sn, K, Na, Mg, Ni, Ca, and V were also found to be contaminants [10, 20, 21, 28, 32–40] (Supplementary Figs. 1 and 2). Ochratoxin A (OA) was also found to be a contaminant [23]. Significant levels of fluoride, chloride, phosphate and sulphate contamination were detected [32, 34, 35] (Table 1) (Supplementary Figs. 3 and 4).

### Sources and quality of water among people with CKDu in Sri Lanka

Forty-three studies were carried out in Sri Lanka assessing the relationship between sources and quality of water and CKDu. They involved the Mullaitivu district (n = 1), Badulla (n = 2), Ampara (n = 2), Monaragala districts (n = 3), all 25 districts in Sri Lanka (n = 2), while the rest were done in Anuradhapura and Polonnaruwa districts in North Central province (NCP) [1, 10, 11, 13–15, 18, 19, 21, 24–26, 28, 29, 32, 37–65]. They included water sources such as wells; dug wells, deep wells, ground wells, tube wells, pipe water, rivers, tanks, reservoirs, irrigation canals, streams and natural springs in their studies. Predominantly they have looked for toxic elements such as As, Cd, Pb, Al, Cr, Co, Cu, Fe, Mn, Zn, Se, Hg, Sn, K, Ni, Antimony (Sb), V, Uranium (U), Lithium (Li), Na, K, Mg, Ca, Boron (B), Silicon (Si), Barium (Ba), Strontium (Sr), Rubidium (Rb), Titanium (Ti), and Molybdenum (Mo) concentrations in these water sources. Cd was the most common contaminant in the water sources demonstrated by the majority of the studies [1, 10, 13–15, 18, 29, 46, 48, 50, 60, 61, 65]. The next major contaminant was As [1, 10, 11, 13, 14, 18, 42, 46, 48, 50, 60, 61, 65]. Third major contaminant was Pb [1, 10, 14, 18, 25, 28, 29, 46, 48, 50, 61, 65]. They also found other contaminants such as Mn, Cu, Fe, Zn, Ni, Cr, Al, Se, Co, Sr, U, Rb, Mo, Li, Ba, V, B, Hg, Sn, Sb, K, Na, Mg, Ca, and Si in the water sources [1, 10, 13, 18, 21, 24, 25, 28, 29, 37, 42, 45, 46, 51, 59, 60, 61, 64, 65] (Supplementary Figures 2, 6 and 7).

These authors also looked for toxic cations such as As^3+^, Cd^2+^, Pb^2+^, Cu^2+^, Zn^2+^, Cr^3+^, Mn^2+^, Fe^2+^, Fe^3+^, Hg^2+^, Li^+^, K^+^, Na^+^, Mg^2+^, Ca^2+^, Sr^2+^, Ba^2+^ and NH_4_^+^ [11, 32, 37, 46, 50, 54, 55, 59, 60, 63, 64] (Supplementary Figures, 8 and 9). Major anions reported in these studies were F^–^, Cl^−^, Br^−^, NO_3_^−^, NO_2_^−^, SO_4_^2−^, PO_4_^3−^, CO_3_^2−^, HCO_3_^−^, NO3^–^ -N, NH_4_^+^-N, and SiO_2_ concentrations in the water sources [10, 11, 13, 18, 21, 24, 25, 29, 37, 44, 45, 46, 48, 50, 51, 54, 55, 58–65] (Supplementary Figures,10 and 114). F^–^ was the predominant anion [10, 11, 13, 18, 21, 24, 25, 32, 37, 44–46, 51, 55, 58–65]. Some studies reported the presence of agrochemicals and fertilizers [42], organic phosphorous [24], glyphosate [49], Aminomethylphosphonic acid (AMPA) [1, 49], 2,4– dichlorophenoxyacetic acid (2,4-D) [53], Pentachlorophenol/ pesticide (PCP) [53], Propanil (PRP) [53], and 3,4–dichloroaniline (3,4-DCA) [53]. These studies also assessed the physicochemical characteristics of water samples such as pH, electrical conductivity, alkalinity, hardness, total dissolved solids (TDS), Eh values, Dissolved Oxygen concentration (DO), Dissolved organic carbon (DOC), Chemical oxygen demand of Permanganate (COD_Mn_), COD_Mn_/DOC ratio, Total Organic Carbon (TOC), turbidity, salinity, temperature, Stable Hydrogen isotope (δ^2^H) (‰), Stable Oxygen isotope (δ^18^O) (‰), Stable Carbon isotope measurements of Dissolved Inorganic Carbon (δ^13^C_DIC_) (‰) and total coliform level as well, [11, 13, 18, 21, 24, 25, 32, 37, 45–48, 50, 51, 53, 55, 59–65] (Supplementary Figs. 12–18). There were two studies reporting the Eh value of the water sources [18, 65] (Table 2).

Research gaps, weakness, and inconsistencies in the literature on consumed food and water quality and sources among people with CKDu in Sri Lanka are reported in Appendix 3.

### Quality of included studies

**Fig. 2:**
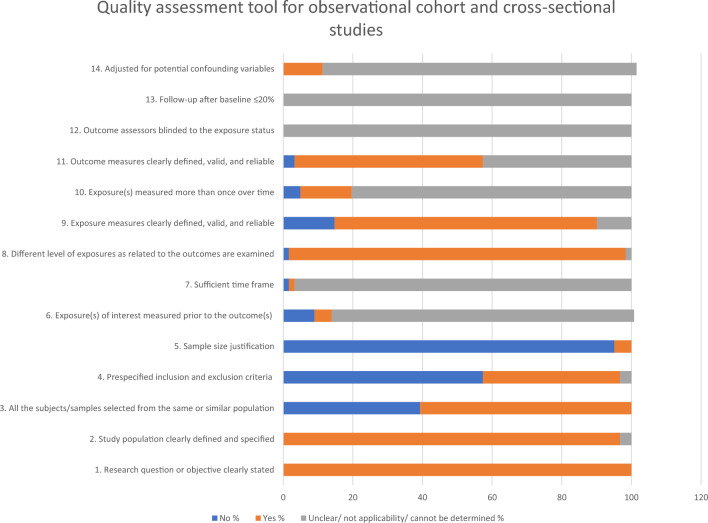
Quality assessment findings of the included studies

In all the studies included in this review, the research question or objective was clearly stated. In most of the studies the study population was clearly defined and specified. In more than half of the studies predefined inclusion and exclusion criteria were not reported. In about 50% of the studies exposure and outcome measures were clearly defined, valid, and reliable. Different exposure levels associated with the outcome were assessed in almost all the studies. However, the majority of studies did not report the sample size justification. Exposure(s) of interest measured prior to the outcome(s), sufficient time frame, exposure(s) measured more than once over time, outcome assessors blinded to the exposure status, follow-up after baseline ≤ 20%, and adjusted for potential confounding variables criteria were not applicable for most of the studies as most of them were cross-sectional studies (Fig. 2).

## Discussion

This systematic review explored patterns in (1) sources and quality of the food consumed (2) sources and quality of water consumed (3) nutritional status, and (4) evidence gaps in the literature on the sources and quality of consumed food and water, and nutritional status of people with CKDu in Sri Lanka.

We found that consumed food including rice, other cereals (non-rice), bread, animal source food, eggs, freshwater fish, fruit and vegetables was contaminated with Cd, Pb, As, Mn, Zn, Cu, Fe, Cr, Se, Co, Al, Hg, Sn, K, Na, Mg, Ni, Ca, and V metals. Water sources including wells (dug wells, deep wells, ground wells, and tube wells), pipe water, rivers, tanks, reservoirs, irrigation canals, streams and natural springs were reported as contaminated with Cd, As, Pb, Mn, Cu, Fe, Zn, Ni, Cr, Al, Se, Co, Sr, U, Rb, Mo, Li, Ba, V, B, Hg, Sn, Sb, K, Na, K, Mg, Ca, Ti, and Si metals. Given the high percentage of contaminants, the impact of agrochemicals and fertilizers in CKDu development should be further researched. Rice and vegetables cultivated in the study areas were found to be contaminated with nephrotoxic heavy metals [14, 15, 24]. Inland fish consumed by the people in the study areas were found to be contaminated with heavy metals such as Cd and this was associated with the CKDu prevalence rate [14].

The water sources investigated in the study areas were contaminated with heavy metals, agrochemicals, fertilizers, herbicides, glyphosate, and AMPA [1, 15, 49]. The recommended levels of these contaminants were exceeded in these water sources, thus posing a high risk for human health [1, 11]. Fluoride levels were also significantly high in some water sources which might be correlated with CKDu in some study areas. High levels of physicochemical properties of water sources also pose health risk [11, 51].

Similar to Sri Lanka, CKDu prevalence is increasing in other South Asian developing countries like India, Bangladesh, Pakistan, Nepal, Bhutan, and Afghanistan [66]. A significant impact on kidney diseases in these countries is caused by environmental pollutants such as heavy metals in the environment leading to increased human exposure due to variety of factors such as contaminated air, drinking water and food [67]. Studies conducted in countries like China, Bangladesh, and Taiwan exploring the positive association between dietary As exposure and estimated GFR (eGFR) support the findings of the current review [68–71]. While Mn, Co, Se, Mo, Pb, and Hg pose health risks to humans, they have also been linked to other diseases such as diabetes, anemia, and to underweight [72]. Modifiable lifestyle factors that are associated with protection from CKD include plant-based diets, sodium restriction, Mediterranean diet, while an increase in risk was reported for dietary Cd intake, red meat, high dietary acid load, high protein diet, and obesity or high BMI [73].

Rice varieties with low Cd concentrations as well as the best fields for paddy cultivation must be identified, and farmers must be educated on measures that reduce Cd concentration in rice and other food [15]. Polished rice is recommended for consumption among vulnerable populations as polishing has been shown to reduce Cd levels [15]. These findings could be applied to the Sri Lankan context to reduce the risk of developing CKDu.

According to quality assessment of the 57 studies included in this review, future studies should focus on justifying the sample size and specifying the inclusion and exclusion criteria in order to improve the methodological quality. Recruitment from the same population will help to reduce the bias in sample selection and increase the methodological quality. However, in almost all the studies, the authors explained the research question or objective clearly and clearly defined and specified the study population. Different exposure levels related to outcome measures were assessed in these studies. Although exposure and outcome measures were clearly defined, valid and reliable in about 50% of the studies, more attention should be paid to further improve the quality of the studies. These quality issues should be considered before generalizing the findings to the population. Future studies are expected to address these limitations, focusing also on recall bias, lack of adjustment for confounders, small sample size, absence of sample size justification, outcome measures, correct measure of the exposures of interests, timeframes, and follow-up.

Future research should include in-depth studies on the impact of glyphosate and AMPA in water and soil on CKDu, on physicochemical properties of water such as hardness, DOC, and should explore the association of nutritional status with the risk of developing CKD.

The current systematic review also has further limitations to acknowledge. The language of the review was limited to English. This may have excluded some publications written in Sinhalese and Tamil, the local languages in Sri Lanka.

In conclusion, this systematic review indicates a higher-level contamination of nephrotoxic heavy metals in the food consumed by people with CKDu in Sri Lanka. Higher levels of Pb, a nephrotoxic heavy metal, were detected in the water sources compared to levels in the consumed food. In addition, high levels of Na^+^ were also found in the studied water sources and highlighted high fluoride levels in water sources consumed by people with CKDu in Sri Lanka. Appropriate strategies to reduce the contamination of heavy metals, agrochemicals, and major ions that reduce the quality of water and food should be implemented to decrease the burden of CKDu in Sri Lanka.

## Appendix 1: Medline search strategy

**Table:** 

#	Searches
1	Renal Insufficiency, Chronic/ or Chronic kidney disease*.mp. or Kidney Failure, Chronic/ or Kidney Diseases/renal disease*.mp
2	chronic kidney disease of unknown cause.mp
3	CKD.mp
4	CKDu.mp
5	CRD.mp
6	CRF.mp
7	CKF.mp
8	1 or 2 or 3 or 4 or 5 or 6 or 7 or 8
9	Water/ or water.mp. or Water Quality/ or Drinking water/
10	(water quality or water resource*).mp
11	concept word, protocol supplementary concept word, rare disease supplementary concept word, unique identifier, synonyms
12	food intake.mp
13	diet.mp. or Diet/
14	diet intake.mp
15	diet* pattern*.mp
16	“Diet, Food, and Nutrition”/ or food.mp
17	food contamination.mp. or Food Contamination/
18	water contamination.mp
19	ground water.mp. or Groundwater/
20	water chemistry.mp
21	Nutritional Status/ or nutrition level*.mp
22	nutrition*.mp
23	10 or 11 or 12 or 13 or 14 or 15 or 16 or 17 or 18 or 19 or 20 or 21 or 22
24	Sri Lanka.mp. or Sri Lanka/
25	Asia, Southeastern/ or Asia/ or Asia.mp
26	South*East*Asia*.mp
27	24 or 25 or 26
28	9 and 23 and 27

## Appendix 2: BMI status of the people with CKDu in Sri Lanka

**Table:** 

First author & year	Geographical location	Type of study	Sample size	Sex	Age (Years)	BMI (kg/m^2^)	Findings
Jayatilake (2013)	E: Anuradhapura, Polonnaruwa and Badulla NE: Hambantota	Cross-sectional	5027	M: 2131 F: 2896	Cases: 39.1 ± 14.2 Con: 39.6	21.7	NR
Nanayakkara (2013)	Medawachchiya and Girandurukotte	Survey	597	M: 597	Cases: 46.6 Con: 41.1	22.05	NR
Siriwardhana (2014)	Medawachchiya	Survey	200	M: 118 F: 82	Cases: 47.8 ± 9.6 Con: 47.7 ± 9.2	22.6	BMI of CKDu patients was closer to the lower limit of normal
Rango (2015)	Anuradhapura, Polonnaruwa, Kandy, Badulla, Ampara and Vavuniya	Survey	109	M: NR F: NR	Cases: 37.5 ± 16.6 Con: 37.5 ± 16.6	21.3	Among the participants 53.7% (n = 72); normal weight or 25.4% (n = 34); underweight. Twenty percent (n = 27) of respondents were overweight, and only one was obese
Siriwardhana (2015)	Medawachchiya	Survey	200	M: 118 F: 82	Cases: 47.8 ± 9.6 Con: 47.7 ± 9.2	22.6	NR

*BMI* Body mass index, *CKDu* Chronic kidney disease of uncertain etiology, *CON* Control, *E* Endemic area, *FE* Females, *M* Males, *NE* Non-endemic area, *NR* Not reported

## Appendix 3: Research gaps in the evidence on sources and quality of the food and water intake among the people with CKDu in Sri Lanka

**Table:** 

Gaps/ Weakness/ Inconsistency	Research gaps/ Future research
Sample size calculation was not statistically justified [8, 11, 19–21, 23–28, 30, 34–36, 38–42, 44–48, 53, 54, 58, 61–65, 74] No predefined inclusion and exclusion criteria [5, 11, 18–20, 24, 29, 31, 32, 34–36, 44, 46, 52, 53, 61–65, 75, 76] Small sample size [23, 24, 27, 32, 44, 48, 51, 57–59] A structured interviewer-administered questionnaire was used to collect information, which is prone to recall bias of the participants [33, 41, 47, 60] Sample size was not clearly specified [13, 15, 42, 49, 50, 60] Multiple exposures were not considered [15, 25, 27, 45] Exposure measures are not valid and reliable [15] Average food consumption pattern considered in this study is not valid and reliable [15] Sample size was limited and not directly linked to consumption habits [25] Failure to quantify the carbon-containing functional groups of both HDOC and SHA by liquid phase ^1^ H and ^13^C NMR [56] Samples from non-CKDu areas were not used [55, 57] Absence of reliable scientific reports on As levels in the reservoir fish species in Sri Lanka for the comparison of study results [31]	Further investigations should focus on environmental factors and on the role of genetic factors [41, 48] Further investigations on other aetiological factors of epidemic kidney disease [8, 23] In-depth studies on the fate of environmental contaminants in soil–water–plant systems in the CKDue region [10] Genetic epidemiology studies on CKDu [10] Further studies to investigate the contributory role of infections in the pathogenesis of CKDu [14] Further studies on individual and combined effects of the metal parameters on CKDu [38] In-depth dietary studies which include the quantitative analysis of dietary intake, total energy and protein intake of the CKDu affected and non-CKDu affected subjects and testing of associated metals in the foods they consume are required [40] Multifactorial studies assessing all the risk factors associated with CKDu [40] Cohort study of the urinary excretion of cadmium and suitable biomarker of kidney damage after appropriate intervention to lower the cadmium intake [15] Future study on food consumption pattern and total exposure to the possible nephrotoxins [15] Relative risk of each drinking and dietary contaminants, should be assessed further [24] Future studies on interactions among concentrations of As, Cd, Pb, and U and whether concentrations of these elements vary across seasons are needed [18] Cohort studies with larger sample sizes encompassing both endemic and non-endemic areas will be required [18] Studies incorporating other measurements associated with hydration status of subjects, including environmental temperature, level of physical activity, hydration efforts during work, awareness regarding the effect of hydration, weight change before and after work, and involving larger groups of farmers and maintaining the authentic setup at their work with least interference needed [39] Animal trials to prove the presence of the fluoride and Aluminium in drinking water for the prevalence patterns of CKDu incidence [13] Investigation of the effect of aluminofluoride complex in CKDu [13] Animal trials to confirm possible link between CKDu to combined effects of high fluoride and high hardness [13] Subsequent study considering additional exposure routes and media [25] A mass scale multi-centered interventional study to access the effect of drinking water sources on the disease progression and generalizing data to the entire CKDu population [48] In-depth studies investigating the possible impacts of glyphosate and AMPA present in water and soils on higher rates of morbidity and mortality due to CKDu [49] A cohort study on the study population to monitor their exposure to these contaminants and their effects using suitable biomarkers and other methods is suggested [27] Future studies focus on food contaminants in the area and corresponding Pb and Cd levels in suitable biological samples of the study population coupled with suitable biomarkers [27] Future studies including study area with a greater number of samples [51] Further investigation to confirm the involvement of As and Cd in pathogenesis of CKDu [28] Further studies to investigate on the sources of Cd and Cr [29] Further research on how to reduce the contaminants in rice and food [30] A research to evaluate complex organic mixtures present in these drinking waters with respect to kidney toxicity [1] Further investigation of the biological effects of glyphosate and metals are needed to interpret these possible molecular interactions [1] Histopathological investigation for the validation purposes about the use of arsenic rich water Kidney Disease: Improving Global Outcomes [11] Future studies to explore the synergistic involvement of hardness and other dissolved ions in the onset of CKDu, through laboratory and field experiments [55] Further research is required to identify the threshold of tolerance for fluoride exposure to establish safe drinking water fluoride concentration for CKDu patients [58] Further investigations to understand how Si is affecting the quality of the groundwater in CKDu affected regions [59] Systematic study of both the human gut and drinking water microbiomes in CKDu patients, in relation to disease prevalence and progression is required [56] To estimate total Hofmeister ion consumption in CKDu endemic regions, it is essential to consider all resources of ions, such as drinking water, drinks, cooked rice, and vegetables [34] Regular monitoring and assessment of groundwater quality is crucial for developing management strategies tomprevent pollution and provide safe drinking water [62] Further research is needed to better understand the impact of CKDu on nephrotoxicity, including non-endemic area studies, dry season analysis, and consideration of biochemical and ion-solvation effects associated with protein denaturation rather than electrochemical ionic strength [63] More research is needed to understand the impact of seasonal precipitation on groundwater geochemistry and the difference in chemical and mineral compositions between CKDu and non-CKDu aquifer sediments [65] The data reported in this study is based on rice taken from a small organic farm and should not be extrapolated to the Anuradhapura region, especially for farms with excessively fertilised or polluted soils [36]

## Supplementary Information

Below is the link to the electronic supplementary material.

Supplementary file1 (DOCX 112 KB)

## Data Availability

Data are available from the first author on request.

## Abbreviations

AMPA: Aminomethylphosphonic acid
Al: Aluminium
As: Arsenic
As^3+^: Arsenic ion
B: Boron
Ba: Barium
Ba^2+^: Barium ion
BMI: Body Mass Index
Br^−^: Bromide
Ca: Calcium
Ca^2+^: Calcium ion
Cd: Cadmium
Cd^2+^: Cadmium ion
CKD: Chronic Kidney Disease
CKDu/ CKDue: Chronic Kidney Disease of unknown etiology
CKF: Chronic Kidney Failure
Cl^−^: Chloride
Co: Cobalt
CO_3_^2−^: Carbonate
COD: Chemical oxygen demand
COD_Mn_: Chemical oxygen demand of Permanganate
CON: Control
CR: Cooked rice
Cr: Chromium
Cr^3+^: Chromium ion
CRD: Chronic Renal Disease
CRF: Chronic Renal Failure
Cu: Copper
Cu^2+^: Cupric ion
2,4-D: 2,4-Dichlorophenoxyacetic acid
3,4-DCA: 3,4-Dichloroaniline
DL: Detection limit
DO: Dissolved Oxygen concentration
DOC: Dissolved organic carbon
DS divisions: Divisional secretary’s (DS) divisions
E: Endemic area
EC: Electrical conductivity
eGFR: Estimated glomerular filtration rate
Eh value: Redox potential value
EMBASE: Excerpta Medica database
ERP: Eppawala rock phosphate
F^−^: Fluoride
FE: Females
Fe: Iron
Fe^3+^: Ferric ion
Fe^2+^: Ferrous ion
GFR: Glomerular Filtration Rate
g/L: Grams per liter
GW: Ground water
HCO_3_^−^: Bicarbonate
HDOC: Protonated dissolved organic carbon
Hg: Mercury
Hg^2+^: Mercuric ion
HR: High risk area
K: Potassium
K^+^: Potassium ion
KDIGO: Kidney Disease Improving Global Outcomes
kg/m^2^: Kilogram per square meter
Li: Lithium
Li^+^: Lithium ion
LR: Low risk area
M: Males
MEDLINE: Medical Literature Analysis and Retrieval System Online
Mg: Magnesium
Mg^2+^: Magnesium ion
µg/L: Micrograms per liter
µg/kg: Micrograms per kilogram
mg/kg: Milligrams per kilogram
mg/24h: Milligrams per 24 h
mg/L: Milligrams per liter
ml/min/1.73m^2^: Milliliters per minute per 1.73 square meter body surface area
MMU black tea: Miniature manufactured ungraded black tea
Mn: Manganese
Mn^2+^: Manganese ion
Mo: Molybdenum
mp: Concept word, protocol supplementary concept word, rare disease supplementary concept word, unique identifier, synonyms
MS Excel: Microsoft Excel
mV: Millivolt
n: Number of studies
Na: Sodium
Na^+^: Sodium ion
NCP: North Central province
NE: Non-endemic area
NH_4_^+^: Ammonium ion
NH_4_^+^- N: Ammonium nitrogen
Ni: Nickle
NO_3_^−^: Nitrate
NO_3_^−^-N: Nitrate-nitrogen
NO_2_^−^: Nitrite
NR: Not reported
NTU: Nephelometric turbidity unit
OA: Ochratoxin A
Pb: Lead
Pb^2+^: Lead ion
PCP: Pentachlorophenol/ pesticide
PICOS: Participants, Intervention/Exposure, Comparison, Outcome, and Study design
PMTDI: Provisional Maximum Tolerable Daily Intake
PO_4_^3−^: Phosphate
ppm: Parts per million
PRISMA: Preferred Reporting Items for Systematic Reviews and Meta Analyses
PROSPERO: International Prospective Register of Systematic Reviews
PRP: Propanil
PsycINFO: Psychological Information Database
Rb: Rubidium
RC: Raw rice
RevMan: Review Manager (Software for Cochrane reviews)
RO: Reverse osmosis
Sb: Antimony
SD: Standard deviation
Se: Selenium
SHA: Synthetic Humic Acid
Si: Silicon
SiO_2_: Silica
SL: Sri Lanka
SLJOL: Sri Lankan Journals Online Database
SLPWL: Sri Lankan Permissible Water Limit
Sn: Tin
SO_4_^2−^: Sulphate
Sr: Strontium
Sr^2+^: Strontium ion
TDS: Total dissolved solids
Ti: Titanium
TRI: Tea Research Institute
U: Uranium
USA: United States of America
V: Vanadium
WHO: World Health Organization
WQI: Water Quality Index
Zn: Zinc
Zn^2+^: Zinc ion
δ^13^C_DIC_: Stable Carbon isotope measurements of Dissolved Inorganic Carbon
δ^2^H: Stable Hydrogen isotope
δ^18^O: Stable Oxygen isotop
