# Supplementary material for: Quality and sources of food and water consumed by people with chronic kidney disease of unknown etiology in Sri Lanka: a systematic review
Source: J Nephrol. 2025 Feb 26;38(5):1287–311. doi: 10.1007/s40620-024-02174-5 (PMC12289754; doi:10.1007/s40620-024-02174-5)
Supplement: Supplementary file 1 — Supplementary file1 (DOCX 112 KB) [file 40620_2024_2174_MOESM1_ESM.docx]

Figure 1: Toxic elements and their levels in endemic food samples reported by different research studies

Figure 2: Toxic elements and their levels in non-endemic food samples reported by Bandara et al, 2023

Figure 3: Toxic anions and their levels in non-endemic food samples reported by different research studies

Figure 4: Toxic anions and their levels in endemic and non-endemic food samples reported by Bandara et al, 2023

Figure 5: Ochratoxin A levels in the food samples reported by Wanigasuriya et al, 2008

Figure 6: Toxic elements and their levels in the endemic water samples reported by the included studies

Figure 7: Toxic elements and their levels in the non-endemic water samples reported by the included studies

Figure 8: Toxic cations and their levels in the endemic water samples reported by the included studies

Figure 9: Toxic cations and their levels in the non-endemic water samples reported by the included studies

Figure 10: Major anions and compounds and their levels in the endemic water samples reported by the included studies

Figure 11: Major anions and compounds and their levels in the non-endemic water samples reported by the included studies

Figure 12: pH values of the water samples reported by the included studies

Figure 13: Electrical conductivity of endemic water samples reported by the included studies

Figure 14: Alkalinity, total hardness, TDS, DOC, DO and COD_Mn_ of endemic water samples reported in the included studies

Figure 15: Temperature (^0^C) of water samples reported by the included studies

Figure 16: δ2H, δ18O, and δ13CDIC levels in water samples reported by the included studies

Figure 17: Turbidity levels in water samples reported by the included studies

Figure 18: Eh, ORP values of water samples reported by the included studies

Figure 19: BMI status of the participants with CKDu reported by the included studies
